# Relationship between fine particulate matter (PM_2.5_) concentration and risk of hospitalization due to chronic obstructive pulmonary disease: a systematic review and meta-analysis

**DOI:** 10.1186/s12889-023-17093-6

**Published:** 2023-11-13

**Authors:** Mouloud Agajani Delavar, Mohammad ali Jahani, Mahdi Sepidarkish, Saeide Alidoost, Hamed Mehdinezhad, Zeynab Farhadi

**Affiliations:** 1 Infertility and Reproductive Health Research Center, Health Research Institute, Babol University of Medical Sciences, Babol, Iran; 2 Social Determinants of Health Research Center, Health Research Institute, Babol University of Medical Sciences, Babol, Iran; 3 National Center for Strategic Research in Medical Education, Tehran, Iran; 4 Department of Internal Medicine, School of Medicine, Rouhani Hospital, Babol University of Medical Sciences, Babol, Iran

**Keywords:** Hospitalization, Introduction, Particulate Matter, Pulmonary Disease, Chronic Obstructive, Air Pollutants, Humans

## Abstract

**Background:**

Short-term exposure to PM2.5 has been associated with human health risks. However, evidence on the association between short-term exposure to PM_2.5_ and the risk of chronic obstructive pulmonary disease (COPD) remains limited and controversial. This study aimed to specifically assess the relationship between exposure to PM_2.5_ and the risk of hospitalization due to COPD.

**Methods:**

A systematic search was conducted in PubMed, Web of Science, and Google Scholar databases from January 1, 2010 to May 1, 2022. The odds ratio (OR) statistic was calculated as a common measure of effect size. Publication bias was also examined in all eligible studies on COPD hospitalization using funnel plots and Egger’s test, as well as trim-and-fill method for missing studies on COPD hospitalization.

**Results:**

A total of 19 studies were included in this meta-analysis. Random-effects models were plotted to calculate the pooled effect size by measuring OR (χ^2^ = 349.95; df = 18; I_2_ = 94.86%; *P* = 0.007; Z = 2.68; *P* < 0.001). A 10-mg/m^3^ daily increase in PM_2.5_ concentration was associated with a 1.6% (95% CI: 0.4–2.9%) increase in COPD hospitalization. There was no publication bias regarding the association between COPD hospitalization and PM_2.5_ (bias = 1.508; 95% CI: -1.475, 4.491; t = 1.066; *P* = 0.301). The subgroups of age ≥ 65 years and Asian countries were associated with an increased risk of COPD hospitalization. Besides, higher risks were estimated in the subgroups of studies performed in the warm season, case-crossover studies, studies with three lag days, and studies without adjustments for humidity and temperature confounders, with very small heterogeneity.

**Conclusion:**

Evidence suggests that short-term exposure to PM_2.5_ increases COPD hospitalization. Further studies are needed to understand the mechanism of the association between PM_2.5_ and COPD for reducing air pollution, which can be beneficial for COPD patients.

## Introduction

COPD, as a life-threatening condition, is recognized as the third most common cause of mortality worldwide. The COPD death rate is the highest in countries with low-resourced healthcare systems [1]. This respiratory disease occurs when the airways and the lungs become inflamed and damaged due to various factors, such as age, sex, genetic predisposition, infections, exposure to environmental tobacco smoke, and other lung irritants [2–5]. In Iran, the COPD death rate has increased over the last decade [6]. Nearly 90% of COPD-related deaths occur in people under 70 years of age in low- and middle-income countries (LMIC) [7].

Air pollution has become one of the most severe and prevalent environmental concerns worldwide [8]. The risk of COPD is known to increase following exposure to unhealthy particles, especially pollutants [9]. There are generally six types of pollutants in the air with significant negative effects on the health of people: ozone, particles with a diameter of 10 µm (PM_10_), PM2.5, nitrogen dioxide, sulfur dioxide, carbon monoxide, and lead[10]. It is estimated that around seven million people die each year due to particulate matter pollution, including ultrafine particles (PM_0.1_), fine particles (PM_2.5_), and coarse particles (PM_10_) [11–13].

Several studies have reported that PM_2.5_ has greater effects on humans compared to PM_10_ [14–16], as it can be inhaled more deeply into the alveoli of the lungs and result in alveolar inflammation and finally COPD [17]. In 2019, Adeloye et al. conducted a systematic population-based study in 65 countries around the world. Their results showed that the global prevalence of COPD was 10.3% in the age range of 30–79 years [18]. Moreover, Faridi et al. found that long-term exposure to PM_2.5_ accounted for 49,303 deaths in adults (≥ 25 years) in 429 cities of Iran in 2018, imposing a cost of about 12,792.1 million dollars [19].

Although most people understand the severity of air pollution to some extent, by evaluating the concentration of air pollutants and their effects, the intensity of air pollution can be accurately estimated [8]. Two systematic reviews and meta-analyses were conducted in 2016 and 2020. Li et al. and Zhu et al. investigated the relationship between PM2.5 exposure and increased hospitalization and mortality from COPD incidence and exacerbation. Li et al. evaluated studies involving short-term exposure to PM2.5 pollutants; But Zhu et al. evaluated studies that included both long-term and short-term exposure. Considering that the results obtained in both studies were inconsistent; [20, 21]. Therefore, the present study aimed to evaluate the short-term association between PM2.5 exposure and the risk of hospitalization for COPD incidence from January 1, 2010 to May 1, 2022.

## Materials and methods

### Search strategy

Two reviewers performed an initial search based on two Medical Subject Headings (MeSH) keywords, that is, “air pollution” and “COPD”. Besides, equivalents of “air pollution” and equivalents of “COPD” were each combined with the odds ratio (OR), and then, the two keywords and time tag were combined (with “AND”). By using specific tags for each electronic database, a search strategy was developed for each one. The search was conducted in PubMed, Scopus, Web of Science, and Google Scholar databases from January 1, 2010 to May 1, 2022. Studies extracted from the databases were entered into EndNote X8. To avoid missing any studies, a manual search was also carried out.

### Selection of studies

Two reviewers identified eligible studies based on the abstracts and titles. The inclusion criteria were as follows: (1) original studies (not meta-analyses or reviews) investigating the relationship between PM_2.5_ and COPD; (2) studies investigating the relationship between the two main variables quantitatively by measuring the effect size (OR/relative risk [RR]/hazard ratio [HR]/excess relative risk [EER], percentage change/percentage increase/rate ratio, and 95% CIs); (3) time-series and case-crossover designs; and (4) studies investigating the effects of PM_2.5_ caused by outdoor air pollution on COPD (5) studies measuring the minimum effect size at log0 or log01; (6) studies only examining PM2.5 concentration per 10 mg/m^3^.

The exclusion criteria were also quite specific: (1) studies evaluating the effects of PM_2.5_ exposure due to indoor biomass burning, tobacco smoking, solid fuel use, and household air pollution on COPD; (2) previous diagnosis of COPD; (3) not belonging to a specific group, such as children, women, or men; (4) no history of certain diseases, such as pneumonia, asthma, or shortness of breath in patients; (5) no history of occupational exposure (e.g., agricultural exposure); (6) death due to COPD; (7) studies not investigating the relationship between the two main variables by measuring the effect size (OR/RR/HR/EER, percentage change/percentage increase/rate ratio, and 95% CIs); (8) studies investigating the effects of acute exacerbation of COPD (AECOPD); and (9) animal studies.

Two investigators independently extracted data from the selected articles using a data extraction form, the results of which were then compared; agreement was reached through consensus. The following data, if available, were extracted from the included studies: study title, authors, year of publication, design, total period of assessment, confounders, pollutant models, pollution exposure, level of pollution exposure, lag days, age of the population, sex of the population, population size, hospitalization due to COPD, COPD OR, min 95% CI for COPD, max 95% CI for COPD, COPD RR, min CI for COPD, max CI for COPD.

Quality assessment was performed according to the Preferred Reporting Items for Systematic Reviews and Meta-Analyses (PRISMA) and Meta-analysis of Observational Studies in Epidemiology (MOOSE) items. Moreover, publication bias was assessed using Begg's and Egger’s tests. Besides, I^2^ statistic, a quantitative measure of inconsistency, was computed to appraise the statistical heterogeneity across studies. The characteristics of included studies are presented in tables and narrative forms, according to the PRISMA statement. Also, random-effects models were used for the meta-analysis when heterogeneity occurred across studies. Cochran's Q test and I^2^ index were calculated for the assessment of heterogeneity and its extent. Besides, the effect of quality score on the key variables was evaluated, and according to the I^2^ values, data were evaluated in the subgroup analysis or meta-analysis, as appropriate. Additionally, the trim-and-fill method was applied to correct any publication bias, and the sensitivity of review findings was determined using restriction techniques (e.g., quality restriction and design restriction).

The subgroup analysis was conducted for age, high quality score group, and lag days of 0–7 in the lag exposure group (separately or combined) if sufficient data were available. Comprehensive Meta-Analysis Software (CMA) Version 3 was used to measure the pooled effect size. In the literature, the generic inverse variance method has been recommended to synthesize data in non-randomized studies [19, 22]. If any of the studies provided the effect size estimates of the subgroups, the pooled effect size was calculated across the subgroups: this process can be interpreted as a meta-analysis at the level of individual studies [19, 22]. Most studies investigated the acute effects within two days after air quality changes; therefore, the average value was determined with lag days of 0–2. Additionally, CMA Version 3 provided estimates of heterogeneity. Chi-square test was used to evaluate the heterogeneity, and a *P*-value < 0.05 was defined to represent significant heterogeneity.

Moreover, a random-effects model was used when there was significant heterogeneity, while a fixed-effects model was plotted when there was non-significant heterogeneity [20, 23]. Besides, publication bias between studies was assessed using funnel plots. The OR was calculated as a common measure of effect size. The RR effect size statistic was converted to OR, according to a study by Zhang and Yu [21, 24]. The following formula was used to calculate OR:

$$\mathrm{OR}=\mathrm{RR}/\mathrm{HR }([1-\mathrm{P}0]+[\mathrm{P}0\times \mathrm{OR}])$$

where *P0* is the incidence rate of the desired outcome in the non-exposed group. The pooled estimates were defined as significant at a *P*-value less than 0.05 (two-sided).

## Results

**Fig. 1:**
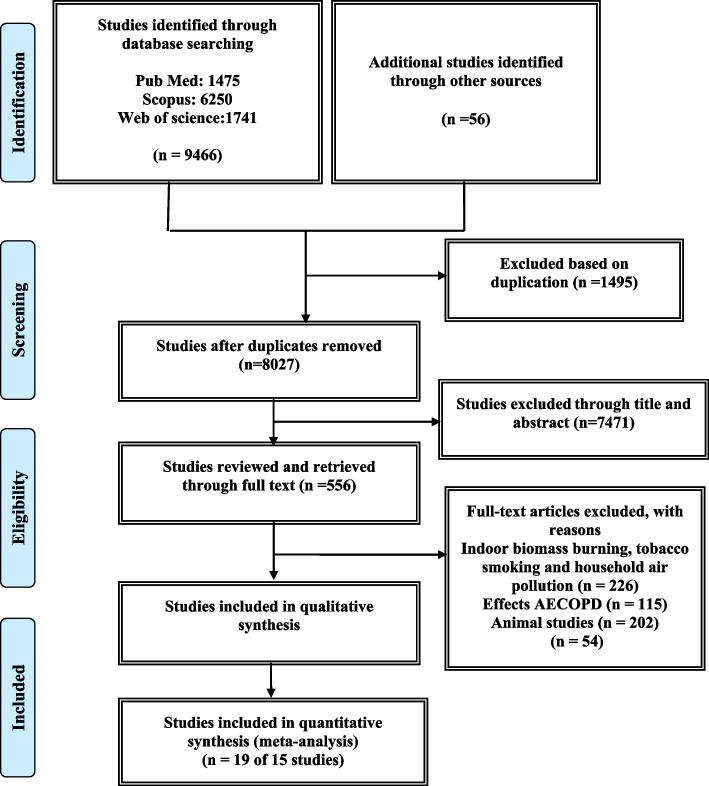
Flow diagram of included/excluded studies

Figure 1 shows 9,466 studies, which were extracted by searching PubMed (*n* = 1475), Scopus (*n* = 6250), and Web of science (*n* = 1741), as well as 52 studies extracted by searching other sources. However, 1495 studies were removed after eliminating duplicates. From the remaining 8027 studies, 7471 studies were excluded by reading the title and abstract, then the full text of 556 studies was read, and those that did not meet the inclusion and exclusion criteria were removed, and finally 15 studies remained. 15 original studies, including 10 time-series analyses and five case-crossover studies, were selected, which met all the inclusion criteria of this review. It should be noted that the findings of a time-series analysis from two different countries were considered as two separate studies. Also, the results of a case-crossover study from four regions of Australia were considered as four separate studies. Table 1 presents the baseline information reported in 19 studies from January 1, 2010 until May 1, 2022.

**Table 1:** Studies included in the meta-analysis investigating for the relationship between PM2.5 and COPD hospitalizations

Author/year	Study location	Study design	Total period measured/month	Pollution exposure	Lag days	Ages	Confounders
Belleudi, 2010 [25]	Rome/Italy	TS	2001–2005/57	PM2.5, PM10, Particle number concentration	0,1,2,3,4,5,6,01,02,05,06	35–64 65–74 ≥ 75	WD
Qiu, 2012 [26]	China/Hong Kong	TS	200–2005/72	PM10 PM2.5, PMc NO2, SO2, O3	0,1,2,3,4,5,01,02,03	All	TT,T,H, I,OAP(NO2,SO2, O3
Kloog, 2014 [27]	United States of America	CC	2000–2006/84	PM2.5	01	≥ 65	T
To, 2015 [28]	Canada/Ontario	TS	2003–2010/96	NO2 O3 PM2.5	0	All	A,G,WD,T,SE,Y,SS,RR
Lopez-VillarrubiaL/P, 2016 [29]	L/P de Gran Canaria	TS	2001–2005/60	PM2.5,PM10-2.5, O3, NO2, SO2, CO	01	All	TT,WD,H,UE,T,RH,BP,I
Lopez-VillarrubiaS/C, 2016	S/C de Tenerife Canaria	TS	2001–2005/60	PM2.5,PM10-2.5, O3, NO2, SO2, CO	01	All	TT,WD,H,UE,T,RH,BP,I
Chang, 2017 [30]	Taiwan/Taipei	TS	2015–2012/49	PM2.5, NO2, SO2	0,1,2,3,4,5,6,7,8,9,10	All < 75 > 75	T
Tian, 2018 [31]		TS	2010–2012/30	PM2.5	0,1,2,3,01,02,03	All < 65 > 65	H,WD,RH,T
Johnstonb, 2019 [32]	Australian/Tasmania, Victoria, New South Wales	CC	2009–2014/72	PM2.5	0,1,2	All	W.M,LT
JohnstonbTas, 2019	Australian/Tasmania	CC	2009–2014/72	PM2.5	0,1,2	All	W.M,LT
JohnstonbVic, 2019	Australian/Victoria	CC	2009–2014/72	PM2.5	0,1,2	All	W.M,LT
JohnstonbNew, 2019	Australian/New South Wales	CC	2009–2014/72	PM2.5	0,1,2	All	W.M,LT
Hopke, 2019 [33]	United States of America/New York	CC	2005–2016/144	PM2.5	0,01,02,03,04,05,06	≥ 18	LT,M,UMC,Se
Bao, 2020 [34]	Lanzhou/ China	TS	48	PM2.5, PMC,	1,2,3,4,5,6,7,01,02,03,04,05,06,07	All < 65 > 65	TT,WD,RH,T,H
Raji, 2020 [35]	Iran/Ahvaz	TS	120	O3, NO, NO2, SO2, CO, PM10, PM2.5	0,1,2,3,4,5,6,7	All	Se,T&RH, WD
Priyankara, 2021 [36]	Sri Lanka/Kandy	TS	2019/12	PM2.5, PM10	0, 1, 2, 3, 4, 5	All	WD, H
Capraz, 2021 [37]	Turkey/Istanbul	TS	2013–2017/59	PM10, PM2.5, NO2	0,1,2,3,4,5,6,7,8,9	All	T, RH
Jin, 2022 [38]	China/Guangzhou	CC	2014–2015/24	PM2.5	0,1,2,3,4,5,6,01,02,03,04,05,06	All	T, RH, H
Haung, 2021 [39]	Taiwan/Kaohsiung	CC	2007–2010/48	PM10, PM2.5, Nitrate, Sulfate, Organic carbon, Elemental carbon	0,1,2,3	All < 65 > 65	T.RH

Study Design: *TS* Time-Series, *CC* Case Crossover, Confounders: *G* Gender, *A* Age, *Se* Seasons, *S* Sex, *OAP* Other Air Pollution, *TT* Time Trend, *W(T&H)* Weather(Temperature,& Humidity), *WD* Weekdays, *H* Holidays, *S-RI* Self-Reported Influenza, *RH* Relative Humidity, *DM* Daily Meteorology, *AA-SP* Annual Age-Specific Population, *ESS* Education Smoking Status, *Y* Year, *BMI* Body Mass Index, *DS* Drinking Status, *PA* Physical Activity, *DM* Diabetes Mellitus, *SS* Socioeconomic Status, *RR* Region of Residence, *LV* Lifestyle Variables, (S,N,A,C,AB) (Sociodemographics, Neighbourhood, Anthropometrics, Comorbidities, Anhaematological Biomarkers), (H,H) (Hypertension, Hyperlipidemia), *MF* Meteorological Factor, *LT* Long Trend, *M* Monthly, *UMC* Underlying Medical Condition

In this study, random-effects models were plotted to determine the pooled effect size by calculating OR (χ^2^ = 349.95; df = 18; I_2_ = 94.86%; *P* < 0.001; Z = 2.68; *P* = 0.007). The random pooled effect size (OR) for the relationship between COPD hospitalization and PM_2.5_ was 1.016 (95% CI: 1.004–1.029). A 10-mg/m^3^ daily increase in PM_2.5_ was associated with a 1.6% increase (95% CI: 0.4–2.9%) in COPD hospitalization (Fig. 1).

Table 2 presents the subgroup analysis of the relationship between PM_2.5_ and COPD hospitalization. All studies showed significant associations between these two variables (*P* < 0.05). The subgroup analyses by age, total period of assessment (short and long), and study location (Asian and non-Asian countries) did not reduce the heterogeneity across studies. However, several characteristics of these studies, including their design, season of analysis, lag days, and adjustments for humidity and temperature, contributed to heterogeneity across eligible studies in this meta-analysis.

**Table 2:** Subgroup analysis for the relationship between PM2.5 and COPD hospitalizations

Group	Studies	Number of Studies	OR(95%CI)	*P*-value	Heterogeneity		
					**x**^**2**^	**I**^**2**^	***P*-value**
**Study location**							
Asian	[31, 34–38]	5	1.008 (1.005,1.010)	< 0.001	218.34	98.16%	< 0.001
Non-Asian	[25–30, 32, 33, 39]	14	1.006 (1.003, 1.010)	< 0.001	130.46	90.03%	< 0.001
**Study design**							
Case-crossover	[27, 32, 33, 38, 39]	8	1.014 (1.010, 1.018)	< 0.001	3.97	0.00%	0.087
Time-series	[25, 26, 28–31, 34, 36, 37, 35]	11	1.005 (1.003, 1.007)	< 0.001	329.87	96.97%	< 0.001
**Season**							
Cold season	[25, 30, 39]	3	1.033 (1.006, 1.061)	0.017	3.758	46.78%	0.153
Warm season	[25, 30, 39]	3	1.037 (1.003, 1.074)	0.035	1.600	0.00%	0.449
**Lag days**							
0 day	[25, 27–29, 31–39]	17	1.007 (1.005–1.009)	< 0.001	321.751	95.02%	< 0.001
1-day	[25, 34, 38, 39]	4	1.005 (1.000–1.009)	0.034	9.446	68.241%	0.024
2- days	[25, 34, 37–39]	4	1.006 (1.001–1.010)	0.101	5.437	44.824%	0.142
3-days	[25, 34, 37–39]	4	1.005 (1.001–1.010)	0.017	0.421	0.000%	0.936
0–1	[25, 34, 37, 38]	4	1.009 (1.004–1.013)	< 0.001	6.696	55.194%	0.082
0–2	[25, 34, 38]	3	1.008 (1.003–1.014)	0.004	1.934	0.000%	0.380
0–3	[25, 34, 38]	3	1.010 (1.004–1.016)	0.002	1.120	10.686%	0.290
**Confounders**							
Humidity and temperature	[26–32, 34, 35, 37–39]	16	1.007 (1.005, 1.009)	< 0.001	346.628	95.67%	< 0.001
Others	[25, 33, 36]	3	1.012 (1.005, 1.019)	0.001	1.228	0.00%	0.0541
**Ages**							
All ages	[25–31, 31–39]	18	1.007 (1.005–1.009)	< 0.001	325.721	94.781%	< 0.001
≥ 65 years	[31, 34, 39]	3	1.030 (1.023–1.036)	< 0.001	97.503	97.949%	< 0.001

In various study designs, time-series analyses reported a 5% higher risk of short-term exposure due to COPD hospitalization; the heterogeneity was non-significant. Also, regarding different seasons (cold and warm), the pooled risk was 1.033 in the cold season and 1.037 in the warm season due to higher PM_2.5_ exposure; the heterogeneity between studies was non-significant. Adjustments for confounding variables, except humidity and temperature, resulted in a 12% increase in the risk of COPD hospitalization due to short-term exposure; the heterogeneity between studies was non-significant. Also, with respect to different lag days, the pooled risk was 6% in two lag days, 5% in three lag days, 9% in 0–1 lag days, 8% in 0–2 lag days, and 10% in 0–3 lag days due to higher PM_2.5_ exposure; the heterogeneity between studies was non-significant.

**Fig. 2:**
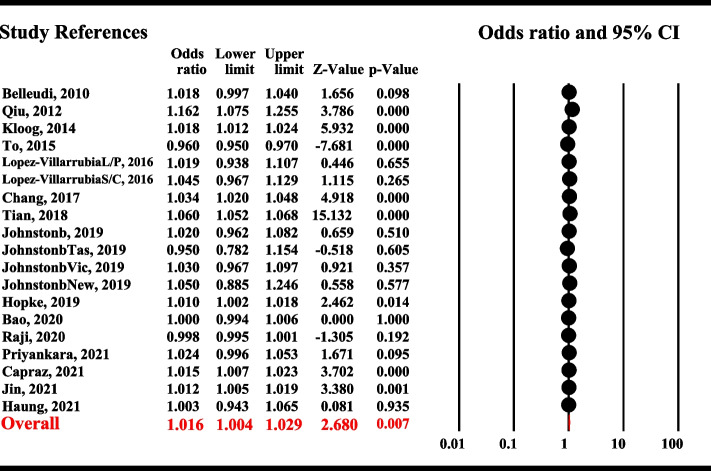
Overall analyses of the effect on the risk of COPD hospitalization associated with a 10 µg/m3 increase in PM2.5

The publication bias was evaluated in all eligible studies using funnel plots for COPD hospitalization. A funnel plot of all the studies did not indicate significant publication bias; almost all study plots focused on the effect size estimate line (Fig. 2). The results of Egger’s test showed no publication bias regarding the relationship between COPD hospitalization and PM_2.5_ (bias = 1.508; 95% CI: -1.475, 4.491; t = 1.066; *P* = 0.301). The trim-and-fill method was also used for missing studies. There were no missing studies on the right side of the mean effect. Nevertheless, according to the trim-and-fill method, six studies were missing on the left side of the mean effect. The adjusted effect size was highly similar to the original effect size (OR = 1.006; 95% CI: 0.993, 1.018; *P* < 0.001).

## Discussion

Exposure to air pollutants has become an important public health concern in the last decade due to its negative impact on people’s emotional state, physical health, mental health, and ultimately well-being. Therefore, attention to air quality standards, set by the World Health Organization (WHO), can significantly reduce mortality due to air pollution. The first step to develop systemic solutions for eliminating or at least reducing the risks is to conduct systematic studies and meta-analyses [10, 11].

In this meta-analysis of 19 studies, the association between PM_2.5_ and the risk of COPD hospitalization was investigated. The results showed that PM_2.5_ was associated with COPD hospitalization. However, pervious meta-analyses reported a slightly stronger positive association between these two variables. A study in 2016 found a 3.1% increase in COPD hospitalization for every 10 mg/m^3^ increase in PM_2.5_ concentration [20]. In two recent meta-analyses (in 2017 and 2018, respectively), every 10 mg/m^3^ increase in PM_2.5_ concentration indicated a 2.5% rise in the risk of COPD hospitalization [21, 39, 40]; therefore, these meta-analyses also showed that PM_2.5_ might increase the risk of COPD hospitalization. Nevertheless, in the present study, there was only a slight increase (1.6% increase) in COPD hospitalization for every 10 μg/m^3^ increase in PM_2.5_ concentration. The current study showed that there was heterogeneity across studies.

In this study, heterogeneity was described in subgroup analyses, the results of which indicated that factors, including the study location, study design, total period of assessment, season of the study, and number of lag days, were possible sources of heterogeneity. Depending on these factors, the association between PM_2.5_ and COPD hospitalization varied. Original studies measured daily variations of PM_2.5_ concentration in a limited geographical area for a limited period of time (0–7 days) and controlled for various confounders [41]. On the other hand, the current study reported different results, and statistical heterogeneity was found for confounding factors and lag days. In accordance with previous research, the incidence, exacerbation and mortality of COPD have been investigated, while the present study specifically addressed the incidence of COPD [21, 42, 43], the present results indicated that the risk of COPD hospitalization was higher in the age subgroup of > 65 years compared with other age subgroups. A possible explanation for this finding may be that elderly people are at a higher risk of the harmful effects of air pollution [44]; however, there are limited studies on PM_2.5_ in the age group of > 65 years.

Consistent with the findings of a study by Moore et al. [45], the present study revealed that the association between PM_2.5_ and COPD hospitalization was stronger in Asian countries, which might be attributed to the fact that the highest concentrations of PM_2.5_ can be found in these countries compared with North America and Europe, according to the WHO guidelines [45]. Similarly, Zhu et al. [21] reported a higher PM_2.5_ risk estimate in case-crossover studies compared with time-series analyses, and the heterogeneity reduced significantly (*I*^*2*^ = 0.00%). In the present study, a higher risk estimate was found in the warm season compared with the cool season, and the heterogeneity reduced significantly (*I*^*2*^ = 0.00%). These results support previous findings, which showed the stronger effect of PM_2.5_ on COPD hospitalization [31]. It has been also suggested that PM_2.5_ concentrations are higher in the warm season compared with the cold season [46].

In the present research, the significant effect of PM_2.5_ on COPD hospitalization was reported in the subgroup of studies controlling for humidity and temperature, as well as studies without adjustments for these factors (despite adjustments for other factors). The effect was greater in the subgroup of studies without adjustments for humidity and temperature compared with studies controlling for those factors, without any heterogeneity (*I*^2^ = 0.00%). In this regard, a previous study showed that PM_2.5_ is strongly linked to weather-related variables, such as temperature, Precipitation, relative humidity, and wind conditions [47].

There were several limitations in this meta-analysis. First, the number of selected studies was limited, and publication bias was not completely assessed. Second, diagnosis of individuals with COPD and hospital administration were according to the International Classification of Diseases External (ICD-9 or ICD-10) or the patient’s symptoms; the diagnosis of COPD was not based on spirometry, which could lead to biased or distorted results. Third Log 0 and 01 have been used to estimate pooled analysis and this issue can be controversial. Fourth Finally, the included studies provided retrospective data of multiple hospitals, where risk factors, such as exposure to indoor air pollutants and smoking habits, were not controlled. It should be also noted that information on the outcomes and exposure to PM_2.5_ was reported by the Governmental Environmental Protection Department. Finally, non-randomized studies are prone to bias, which is difficult to identify and deal with, and even the Handbook Review of Cochrane has not yet provided analytical methods for this category of studies [48, 49].

## Conclusion

The results of this systematic review and meta-analysis indicated that short-term exposure to PM_2.5_ might increase the risk of COPD hospitalization. The subgroups of age ≥ 65 years, Asian countries, warm season, case-crossover design, and three lag days were risk factors for the adverse effects of PM_2.5_. Also, higher risks were estimated in the subgroup analyses. Various other confounders, such as humidity and temperature, produced variable results. Therefore, further studies are needed to understand the mechanism of the association between PM_2.5_ and COPD for developing preventative strategies and improving air quality to reduce the concentrations of air pollutants. The development of transportation and public health policies to control PM2.5 at the global standards will reduce the incidence of COPD. The present study provides evidence that the time has come to take necessary measures and continuous assessment to improve air quality.

The study protocol was registered in the International Prospective Register of Systematic Reviews (PROSPERO) on June 6, 2022 (ID: CRD42022338348).

### Patient and public involvement

Considering that the study was conducted in a systematic review method; Patients were not involved. I thank all the authors of the studies included in this research.

## Data Availability

All data generated or analyzed during this study are included from preliminary studies are available from the corresponding author on reasonable request.

## References

[1] Soriano JB, Kendrick PJ, Paulson KR, Gupta V, Abrams EM, Adedoyin RA (2020). Prevalence and attributable health burden of chronic respiratory diseases, 1990–2017: a systematic analysis for the Global Burden of Disease Study 2017. Lancet Respir Med.

[2] World Health Organization. Causes Chronic obstructive pulmonary disease(COPD).review due: 20 September 2022. Available at: https://www.nhs.uk/conditions/chronic-obstructive-pulmonary-disease-copd/causes/. November 29, 2022.

[3] Lortet-Tieulent J, Soerjomataram I, López-Campos JL, Ancochea J, Coebergh JW, Soriano JB. International trends in COPD mortality, 1995–2017. Eur Respir J. 2019;54(6). 10.1183/13993003.01791-2019 31744832

[4] Postma DS, Bush A, van den Berge M (2015). Risk factors and early origins of chronic obstructive pulmonary disease. Lancet.

[5] Alahmad B, Al-Hemoud A, Kang C-M, Almarri F, Kommula V, Wolfson JM (2021). A two-year assessment of particulate air pollution and sources in Kuwait. Environ Pollut.

[6] Pishgar E, Fanni Z, Tavakkolinia J, Mohammadi A, Kiani B, Bergquist R (2020). Mortality rates due to respiratory tract diseases in Tehran, Iran during 2008–2018: a spatiotemporal, cross-sectional study. BMC Public Health.

[7] World Health Organization.Chronic obstructive pulmonary disease (COPD). 16 March 2023. Available at:https://www.who.int/news-room/fact-sheets/detail/chronic-obstructive-pulmonary-disease(copd)#:~:text=Nearly%2090%25%20of%20COPD%20deaths,%2Dincome%20countries%20(LMIC).&text=Tobacco%20smoking%20accounts%20for%20over,is%20a%20major%20risk%20factor.

[8] Li C, Managi S (2022). Spatial variability of the relationship between air pollution and well-being. Sustain Cities Soc.

[9] Zielinska MA, Hamulka J (2019). Protective effect of breastfeeding on the adverse health effects induced by air pollution: current evidence and possible mechanisms. Int J Environ Res Public Health.

[10] Grzywa-Celińska A, Krusiński A, Milanowski J (2020). ‘Smoging kills’-effects of air pollution on human respiratory system. Ann Agric Environ Med.

[11] Khilnani GC, Tiwari P (2018). Air pollution in India and related adverse respiratory health effects: past, present, and future directions. Curr Opin Pulm Med.

[12] Chen Z, Fu Q, Mao G, Wu L, Xu P, Xu D (2021). Increasing mortality caused by chronic obstructive pulmonary disease (COPD) in relation with exposure to ambient fine particulate matters: an analysis in Southeastern China. Environ Sci Pollut Res.

[13] Zhu F, Chen L, Qian Z, Liao Y, Zhang Z, McMillin SE (2021). Acute effects of particulate matter with different sizes on respiratory mortality in Shenzhen. China Environ Sci Poll Res.

[14] Tsai SS, Chang CC, Yang CY (2013). Fine particulate air pollution and hospital admissions for chronic obstructive pulmonary disease: a case-crossover study in Taipei. Int J Environ Res Public Health..

[15] Pope CA, Dockery DW (2006). Health effects of fine particulate air pollution: lines that connect. J Air Waste Manag Assoc.

[16] Oberdörster G, Oberdörster E, Oberdörster J (2005). Nanotoxicology: an emerging discipline evolving from studies of ultrafine particles. Environ Health Perspect.

[17] Dominici F, Peng RD, Bell ML, Pham L, McDermott A, Zeger SL (2006). Fine particulate air pollution and hospital admission for cardiovascular and respiratory diseases. JAMA.

[18] Adeloye D, Song P, Zhu Y, Campbell H, Sheikh A, Rudan I (2022). Global, regional, and national prevalence of, and risk factors for, chronic obstructive pulmonary disease (COPD) in 2019: a systematic review and modelling analysis. Lancet Respir Med.

[19] Faridi S, Bayat R, Cohen AJ, Sharafkhani E, Brook JR, Niazi S, et al. Health burden and economic loss attributable to ambient PM2. 5 in Iran based on the ground and satellite data. Sci Rep. 2022;12(1):14386. 10.1038/s41598-022-18613-x PMC9399101 35999246

[20] Li M-H, Fan L-C, Mao B, Yang J-W, Choi AM, Cao W-J (2016). Short-term exposure to ambient fine particulate matter increases hospitalizations and mortality in COPD: a systematic review and meta-analysis. Chest.

[21] Zhu R-X, Nie X-H, Chen Y-H, Chen J, Wu S-W, Zhao L-H. Relationship between particulate matter (PM2. 5) and hospitalizations and mortality of chronic obstructive pulmonary disease patients: a meta-analysis. Am J Med Sci. 2020;359(6):354–64. 10.1016/j.amjms.2020.03.016 32498942

[22] Higgins JP, S. G. Cochrane handbook for systematic reviews of interventions Version 5.0.2 [updated September 2009]. The Cochrane Collaboration. Available online: www.cochrane-handbook.org (Accessed on 29 Sept 2009). 2009.

[23] Lin H-H, Ezzati M, Murray M (2007). Tobacco smoke, indoor air pollution and tuberculosis: a systematic review and meta-analysis. PLoS Med.

[24] Zhang J, Kai FY (1998). What's the relative risk?: A method of correcting the odds ratio in cohort studies of common outcomes. JAMA.

[25] Belleudi V, Faustini A, Stafoggia M, Cattani G, Marconi A, Perucci CA, et al. Impact of fine and ultrafine particles on emergency hospital admissions for cardiac and respiratory diseases. Epidemiology. 2010:414–23. 10.1097/EDE.0b013e3181d5c021 20386174

[26] Qiu H, Yu IT, Tian L, Wang X, Tse LA, Tam W (2012). Effects of coarse particulate matter on emergency hospital admissions for respiratory diseases: a time-series analysis in Hong Kong. Environ Health Perspectives..

[27] Kloog I, Nordio F, Zanobetti A, Coull BA, Koutrakis P, Schwartz JD (2014). Short term effects of particle exposure on hospital admissions in the Mid-Atlantic states: a population estimate. PLoS One.

[28] To T, Feldman L, Simatovic J, Gershon AS, Dell S, Su J (2015). Health risk of air pollution on people living with major chronic diseases: a Canadian population-based study. BMJ Open.

[29] López-Villarrubia E, Iñiguez C, Costa O, Ballester F (2016). Acute effects of urban air pollution on respiratory emergency hospital admissions in the Canary Islands. Air Qual Atmos Health.

[30] Chang J-H, Hsu S-C, Bai K-J, Huang S-K, Hsu C-W (2017). Association of time-serial changes in ambient particulate matters (PMs) with respiratory emergency cases in Taipei's Wenshan District. PLoS One.

[31] Tian Y, Xiang X, Juan J, Song J, Cao Y, Huang C (2018). Short-term effects of ambient fine particulate matter pollution on hospital visits for chronic obstructive pulmonary disease in Beijing. China Environ Health.

[32] Johnston FH, Salimi F, Williamson GJ, Henderson SB, Yao J, Dennekamp M (2019). Ambient particulate matter and paramedic assessments of acute diabetic, cardiovascular, and respiratory conditions. Epidemiology.

[33] Hopke PK, Croft D, Zhang W, Lin S, Masiol M, Squizzato S, et al. Changes in the acute response of respiratory diseases to PM2. 5 in New York State from 2005 to 2016. Sci Total Environ. 2019;677:328-39 10.1016/j.scitotenv.2019.04.357 31059876

[34] Bao H, Dong J, Liu X, Tan E, Shu J, Li S (2020). Association between ambient particulate matter and hospital outpatient visits for chronic obstructive pulmonary disease in Lanzhou. China Environ Sci Poll Res.

[35] Raji H, Riahi A, Borsi SH, Masoumi K, Khanjani N, AhmadiAngali K, et al. Acute effects of air pollution on hospital admissions for asthma, COPD, and bronchiectasis in Ahvaz, Iran. Int J Chronic Obstructive Pulmon Dis. 2020:501–14. 10.2147/COPD.S231317 PMC7061718 32184587

[36] Priyankara S, Senarathna M, Jayaratne R, Morawska L, Abeysundara S, Weerasooriya R, et al. Ambient PM2. 5 and PM10 exposure and respiratory disease hospitalization in Kandy, Sri Lanka. Int J Environ Res Public Health. 2021;18(18):9617. 10.3390/ijerph18189617 PMC8466407 34574538

[37] Çapraz Ö, Deniz A (2021). Assessment of hospitalizations from asthma, chronic obstructive pulmonary disease and acute bronchitis in relation to air pollution in İstanbul. Turkey Sustain Cities Soc.

[38] Jin J-Q, Han D, Tian Q, Chen Z-Y, Ye Y-S, Lin Q-X, et al. Individual exposure to ambient PM 2.5 and hospital admissions for COPD in 110 hospitals: a case-crossover study in Guangzhou, China. Environ Sci Poll Res. 2022;29(11699–706). 10.1007/s11356-021-16539-x PMC8794997 34545525

[39] Huang Y-T, Chen C-C, Ho Y-N, Tsai M-T, Tsai C-M, Chuang P-C (2021). Short-term effects of particulate matter and its constituents on emergency room visits for chronic obstructive pulmonary disease: a time-stratified case-crossover study in an urban area. Int J Environ Res Public Health.

[40] DeVries R, Kriebel D, Sama S. Outdoor air pollution and COPD-related emergency department visits, hospital admissions, and mortality: a meta-analysis. COPD. 2017;14(1):113–21. 10.1080/15412555.2016.1216956 PMC8994423 27564008

[41] Sunyer J (2001). Urban air pollution and chronic obstructive pulmonary disease: a review. Eur Respir J.

[42] Sun Q, Liu C, Chen R, Wang C, Li J, Sun J (2019). Association of fine particulate matter on acute exacerbation of chronic obstructive pulmonary disease in Yancheng, China. Sci Total Environ.

[43] Li J, Sun S, Tang R, Qiu H, Huang Q, Mason TG, et al. Major air pollutants and risk of COPD exacerbations: a systematic review and meta-analysis. Int J Chronic Obstruct Pulmon Dis. 2016:3079–91. 10.2147/COPD.S122282 PMC5161337 28003742

[44] Pavord ID, Jones PW, Burgel P-R, Rabe KF (2016). Exacerbations of COPD. Int J Chron Obstruct Pulmon Dis.

[45] Moore E, Chatzidiakou L, Kuku M-O, Jones RL, Smeeth L, Beevers S, et al. Global associations between air pollutants and chronic obstructive pulmonary disease hospitalizations. A systematic review. Ann Am Thoracic Soc. 2016;13(10):1814–27. 10.1513/AnnalsATS.201601-064OC PMC5122486 27314857

[46] Jadidi H, Shahsavani A, Mahaki B. Spatial and Temporal Variations of PM2.5 Concentration and Air Quality in Isfahan City in 2016. J Environ Health Sustain Dev. 2019;4(1):685–93.

[47] Requia WJ, Jhun I, Coull BA, Koutrakis P. Climate impact on ambient PM2. 5 elemental concentration in the United States: a trend analysis over the last 30 years. Environ Int. 2019;131:104888. 10.1016/j.envint.2019.05.082 31302483

[48] Julian PT Higgins, Green. S. Cochrane Handbook for Systematic Reviews of Interventions 4.2.6 Updated September 2006.

[49] Higgins JP, Green S. Cochrane Handbook for Systematic Reviews of Interventions Version 5.0.2. The Cochrane Collaboration. Available online: www.cochrane-handbook.org (Accessed on 29 Sept 2009). 2009.

